# Using macular velocity measurements to relate parameters of bone conduction to vestibular compound action potential responses

**DOI:** 10.1038/s41598-023-37102-3

**Published:** 2023-06-23

**Authors:** Christopher J. Pastras, Ian S. Curthoys, Richard D. Rabbitt, Daniel J. Brown

**Affiliations:** 1 Faculty of Science and Engineering, School of Engineering, Macquarie University, Sydney, NSW 2109 Australia; 2 School of Medical Sciences, The University of Sydney, Sydney, NSW 2050 Australia; 3 Vestibular Research Laboratory, School of Psychology, The University of Sydney, Sydney, NSW 2050 Australia; 4 Departments of Biomedical Engineering, Otolaryngology and Neuroscience Program, University of Utah, Salt Lake City, UT 84112 USA; 5 School of Pharmacy and Biomedical Sciences, Curtin University, Bentley, WA 6102 Australia

**Keywords:** Neuroscience, Physiology, Systems biology, Medical research

## Abstract

To examine mechanisms responsible for vestibular afferent sensitivity to transient bone conducted vibration, we performed simultaneous measurements of stimulus-evoked vestibular compound action potentials (vCAPs), utricular macula velocity, and vestibular microphonics (VMs) in anaesthetized guinea pigs. Results provide new insights into the kinematic variables of transient motion responsible for triggering mammalian vCAPs, revealing synchronized vestibular afferent responses are not universally sensitive to linear jerk as previously thought. For short duration stimuli (< 1 ms), the vCAP increases magnitude in close proportion to macular velocity and temporal bone (linear) acceleration, rather than other kinematic elements. For longer duration stimuli, the vCAP magnitude switches from temporal bone acceleration sensitive to linear jerk sensitive while maintaining macular velocity sensitivity. Frequency tuning curves evoked by tone-burst stimuli show vCAPs increase in proportion to onset macular velocity, while VMs increase in proportion to macular displacement across the entire frequency bandwidth tested between 0.1 and 2 kHz. The subset of vestibular afferent neurons responsible for synchronized firing and vCAPs have been shown previously to make calyceal synaptic contacts with type I hair cells in the striolar region of the epithelium and have irregularly spaced inter-spike intervals at rest. Present results provide new insight into mechanical and neural mechanisms underlying synchronized action potentials in these sensitive afferents, with clinical relevance for understanding the activation and tuning of neurons responsible for driving rapid compensatory reflex responses.

## Introduction

Vestibular otolith organs are phylogenetically ancient inertial sensors that evolved hundreds of millions of years ago in primitive fish^1^, and successfully endowed extant land-dwelling vertebrates with the sensory neural inputs necessary for locomotion and navigation in a complex terrestrial environment^2–5^. In amniotes, some otolith afferent neurons preferentially respond to low-frequency gravito-inertial acceleration^6–11^, while others preferentially respond to high-frequency air conducted sound (ACS) or bone conducted vibration (BCV)^12–19^. The full population of otolith sensory neurons provide the central nervous system with broad-band detection of linear acceleration and head orientation in three-dimensional (3D) space, providing critical inputs to the autonomic nervous system to modulate heart rate and respiration during movements^20,21^, and to motor circuits responsible for the vestibular-ocular, -spinal, and -colic reflexes^9,22^. The compensatory nature of vestibular circuits makes disorders of the otolith organs particularly debilitating, often leading to sensory conflict and symptoms of dizziness, nausea, blurred vision, anxiety, and disorientation. Otolith function is commonly tested in the clinic using transient ACS or BCV to evoke reflexive cervical or ocular myogenic potentials (VEMPs), but precisely how high-frequency transient stimuli lead to mechano-transduction and neural responses in otolith organs is not well understood.

The broad dynamic range of otolith sensitivity from DC to several kilohertz^23^ arises from diverse properties of hair cells, synapses, and vestibular afferent spike generators^24,25^. Amniote neuroepithelia have two major hair cell types (I and II) and two major synaptic terminal types (bouton, calyx, or their combination; dimorphic)^26–28^ which combine with spike generation properties to provide the broad frequency bandwidth and diversity in action potential generation between different afferent neurons. The larger diameter calyx bearing afferents, which evolved in land-dwelling amniotes^2,3,29^, make synaptic contacts with type-I hair cells in the striolar region of the macula^28,30,31^, and are characterized by their irregular action potential discharge rate, phasic responses to maintained stimuli, and sensitive short-latency responses to linear acceleration^32^. Calyx synaptic terminals completely envelop the lateral and basal surface of one or more type-I hair cells and are exquisitely sensitive to transient stimuli^18,33^. Three modes of excitatory synaptic transmission occur at calyx terminals: quantal glutamatergic vesicular release (QT)^34,35^, ultrafast nonquantal ephaptic coupling (NQf)^24,36^, and slow nonquantal accumulation of K^+^ within the synaptic cleft (NQs)^24,37,38^. Direct ephaptic electrical coupling (NQf) is the component responsible for ultrashort latency and high sensitivity of calyx bearing vestibular afferents to transient inputs^36^.

Sensitivity of calyx bearing otolith afferent neurons to transient BCV and ACS is routinely exploited in the clinic and the laboratory to test otolith function. In the clinic, reflexive cervical and ocular vestibular evoked myogenic potentials (cVEMP and oVEMP) are used to test saccular and utricular function^39^, and in the laboratory short latency vestibular stimulus evoked potentials (VsEP) are used to screen otolith function in mice and other rodents^40^. VsEPs are compound action potentials arising from transient stimuli that evoke nearly synchronous firing of a large number of calyx-bearing afferent neurons. When the vestibular compound action potential (vCAP) is recorded from localized sites near the vestibular nerve branch such as the facial nerve canal, the signal-to-noise ratio is enhanced providing recordings similar to auditory CAPs recorded from the round window niche^41,42^. vCAPs reflect combined responses of the population of sensitive afferent neurons and have been recorded in both acute and chronic animal models of health and disease^43^. Although whole-nerve neural responses to transient stimuli have been reported for otolith organs, it is currently not known how high frequency transient stimuli lead to mechano-electrical transduction (MET) by sensory hair cells or the generation of synchronized action potentials.

The present report quantifies the relationship between mechanical vibration of the macula, gating of hair cell MET channels, and generation of vCAPs in the guinea pig utricle for BCV stimuli. This was achieved by simultaneous measurement of temporal bone (linear) acceleration, macular velocity, vestibular microphonics (VMs), and extracellular vCAPs. Results provide new insight into mechanical and receptor mechanisms underlying synchronized neural responses in phasic vestibular afferents, with clinical relevance for understanding vestibular reflex responses, used to diagnose vestibular health and disease at the bedside.

## Methods

### Animal preparation and surgery

Experiments were performed on 28 adult tri-colored guinea pigs (*Cavia porcellus*) weighing between 300 and 500 g of either sex. All experiments performed in this study were approved by the University of Sydney Animal Care and Ethics Committee (Approval number: #2019/1533). All methods were carried out in accordance with the relevant guidelines and regulations, which included the Australian Code for the Care and Use of Animals for Scientific Purposes (8th edition, 2013), and the ARRIVE guidelines^44^. Prior to procedures, animals first received pre-anesthetic intraperitoneal injections of Atropine Sulphate; 0.1 mg/kg (0.6 mg/ml; Apex Laboratories, NSW, Australia) and Buprenorphine Hydrochloride; 0.05 mg/kg (Temgesic; 324 µg/ml; Reckitt Benckiser, Auckland, NZ). Thereafter, animals were anesthetized in an induction chamber with Isoflurane (2–4%; Henry Schein, NSW, Australia) saturated in medical O_2_ (Coregas, NSW, Australia). Once lacking a foot-withdrawal reflex, guinea pigs were transferred to the surgical table, and received anesthetic via a nose cone, whilst local injections of lignocaine hydrochloride (Lidocaine, Troy Laboratories, NSW, Australia) were delivered to surgery sites. Animals were then tracheotomized and artificially ventilated using Isoflurane (~ 2%) with oxygen, with the aid of a small animal ventilator (Model 683, Harvard Apparatus, MA, USA).

### Stimulus delivery

**Figure 1:**
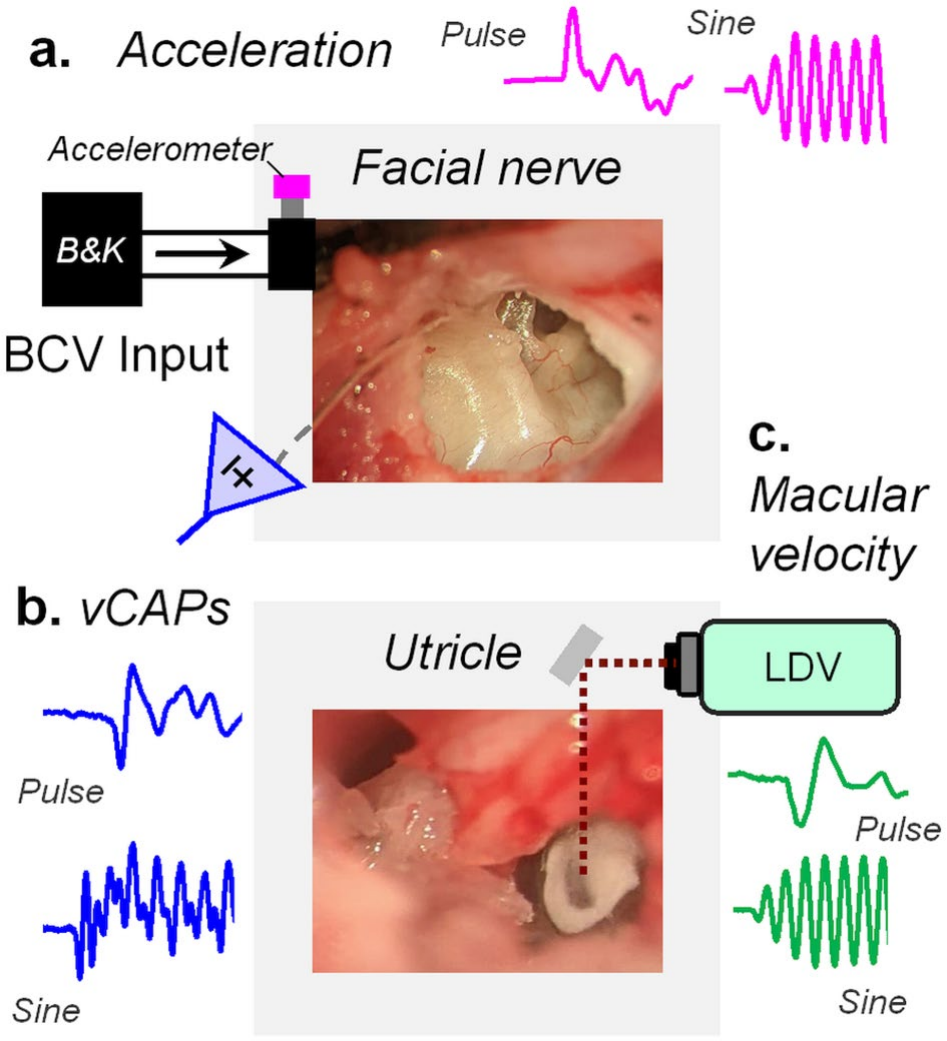
The experimental approach to record vestibular afferent and vibration responses. The guinea pig was mounted in custom-made ear bar frames, and surgery was performed to access the tympanic bulla and sensory end-organs of the labyrinth using a ventral approach. (**a**) Transient pulsatile or sinusoidal tone burst BCV stimuli (magenta) were used to evoke, (**b**) synchronized vestibular Compound Action Potentials (vCAPs) (blue) recorded from the facial nerve canal in anesthetized guinea pigs. (**c**) Simultaneous measurements of macular vibration (green) were measured from reflective microbeads placed on the basal epithelial surface of the utricle via Laser Doppler Vibrometry (LDV).

Guinea pigs were mounted in custom-made ear-bar frames (Thorlabs, NJ, USA). For the delivery of bone-conducted vibration (BCV) stimuli, an electrodynamic minishaker (Type-4810, Brüel & Kjær, Denmark) was attached to the ear-bar in the inter-aural plane via a 5 cm metal rod (Fig. 1).

### vCAP recording

To record the vestibular Compound Action Potential (vCAP), the dorsolateral bulla was exposed and opened via a postauricular surgical approach, with the guinea pig laying supine and mounted in custom-made ear-bars (modular setup using components from Thorlabs, NJ, USA). A single channel two-electrode differential recording montage was used to measure vCAPs. Here, the non-inverting (active) electrode was a fabricated 200 µm Ag/AgCl electrode that was inserted ~ 3 mm into the bony facial nerve canal, near the vestibular branch of cranial nerve VIII (see. Figure 1A). The inverting (reference) electrode was a custom-made Ag/AgCl electrode that was inserted into nearby neck musculature. All biopotentials were grounded via a low-resistance earth electrode placed in the nape of the neck, covered in saline-soaked gauze. vCAPs were evoked by transient pulses or tone-burst stimuli; neural origins were confirmed with chemical ablation of vCAPs following tetrodotoxin (TTX; 100 µM in artificial perilymph; Sigma Aldrich, AUS) (Supplementary Fig. 1, S1).

### VM recording

To record localized Vestibular Microphonic (VM) potentials from the basal surface of the utricular macula, the cochlea was surgically exposed and ablated using a ventral surgical approach, to provide a full view of the utricular macular epithelium under the observation of the operating microscope (see. Figs. 1, 3 and Pastras et al.^59^). The VM was measured using a two-electrode single-ended recording montage. The active electrode was an Ag/AgCl electrode placed into a pulled Borosilicate pipette with a tip diameter of ~ 10 µm and backfilled with 250 mM of NaCl. The pipette was positioned in the vestibule using a manual 3-axis micromanipulator fixed to an isolation table. The pipette electrode was guided down to the surface of the macula until touching the thin layer of perilymph above the epithelium (see. Pastras et al. 2017).

### Laser Doppler vibrometry measurements

A single-point LDV (type 8338—Brüel & Kjær, Denmark) was used to measure the dynamic response of the utricular macula during transient vibration stimulation. LDV output was calibrated against a triaxial accelerometer prior to each experiment (Supplementary Fig. 2, S2). To increase the LDV signal strength, reflective microbeads (20 µm diam., > 1.93 Refractive Index, Cospheric, CA, USA) were positioned on the macula under guidance of a surgical microscope. The LDV laser beam was then directed onto the microbead targets via an adjustable optical mirror in 3D (Thorlabs, NJ, USA) (Fig. 1b). Perilymph build-up over the bead was controlled by the placement of tissue wicks into the vestibule, which minimised artifacts in the LDV recordings due to fluid surface motion effects. When recording vCAP responses, attempts were made to position the bead at the dark band at the centre of the macula, which corresponds approximately to the striolar region (Fig. 1 and Supplementary Fig. 3, S3). However, measures of macular vibration at the lateral striolar region revealed minimal to no differences to that of the central ‘striolar’ zone for pulsatile vibration (Supplementary Fig. 3, S3). This suggested that discrepancies in bead placement across animals did not alter mechanical results based on spatial tuning of the macula.

### Linear acceleration and jerk

A triaxial piezoelectric accelerometer (Model 832M1-0200, TE connectivity, NSW, Australia) with a frequency response of 2-6000 Hz and range of ± 25 g, was mounted to the ear-bar frame adjacent to the skull using a screw thread adapter, in the same plane as the bone-conductor (interaural axis). Linear jerk was calculated by taking the first derivative of acceleration from the ear-bar. Linear acceleration (and its first derivative) was a good proxy for adjacent temporal bone acceleration for all stimuli featured, confirmed experimentally with single-point LDV recordings from the temporal bone in the inter-aural plane, which closely matched adjacent accelerometer values (in the same axis) (Supplementary Fig. 4, S4).

### Stimuli and recordings

Stimuli and responses were generated and recorded using custom-developed LabVIEW programs (National Instruments, TX, USA). BCV stimuli were generated using a high-resolution external soundcard, USB DAC (SoundblasterX7; Creative Inc., Singapore). Analogue responses were amplified by 80 dB (× 10,000), with a 0.1 Hz to 10 kHz band-pass filter (IsoDAM8, WPI, Florida, USA) before being digitized at a rate of 40,000 Hz. All responses were averaged using 100 stimulus presentations.

### vCAP and macular sensitivity with changes in stimulus rise-time

Previous work by Jones et al. investigated the relevant kinematic component of head motion responsible for triggering mammalian VsEPs by varying stimulus rise-times and keeping headframe acceleration (or jerk) constant, whilst varying input jerk (or acceleration)—termed iso-acceleration and iso-jerk, respectively^45^. Results revealed VsEPs scaled with kinematic jerk in their mouse model, evoked by intense dorsoventral accelerations (up to 5G). However, acceleration and jerk were measured by a calibrated accelerometer mounted on an aluminium plate bolted to the electrodynamic shaker piston. Hence, there were no direct measures of cranial acceleration and otolith mechanics. With the ability to measure utricular velocity using LDV, and with larger vestibular nerve compound field potentials recorded closer to the peripheral generators, this study more comprehensively aimed to examine the stimulation sensitivity of the vestibular afferents and utricular macula to clinically relevant transient BCV. In addition to replicating iso-acceleration and iso-jerk measurements, as previously documented in the field^45^, we wanted to further investigate changes in vestibular afferent sensitivity with direct measures of utricular macular velocity related to interaural BCV acceleration. To do this, several stimulation paradigms were used which involved changing the rise-time of the stimulus whilst maintaining fixed peak voltage output driving the minishaker or whilst maintaining a fixed peak level of macular vibration, input linear acceleration, or input linear jerk. Macular velocity was determined by direct LDV measurements, whereas linear acceleration (and jerk) was determined via the ear-bar accelerometer measurements.

These paradigms are hereafter referred to using the following terms:

$${\text{Fixed peak voltage to minishaker }}\left( {\text{changes in rise time}} \right) = {\text{Iso - Stimulus Voltage}}.$$

$${\text{Fixed peak macular velocity}} = {\text{Iso - Macular Velocity}}.$$

$${\text{Fixed peak temporal bone acceleration}} = {\text{Iso - Linear Acceleration}}.$$

$${\text{Fixed peak temporal bone jerk}} = {\text{Iso - Linear Jerk}}.$$

The purpose of these different paradigms was to systematically examine how macular velocity and vCAP response amplitudes changed as stimulus rise-fall time was varied during clinically relevant impulsive BCV stimuli in the mammalian labyrinth.

### Significant statement

Calyx-bearing afferents in the utricle have the remarkable ability to fire an action potential at a precise time following the onset of a transient stimulus and provide temporal information required for compensatory vestibular reflex circuits, but specifically how transient high-frequency stimuli lead to mechanical activation of hair cells and neural responses is poorly understood. Here, we dissect the relative contributions of mechanics, hair cell transduction, and action potential generation on short-latency responses to transient stimuli. Results provide a framework for the interpretation of synchronized vestibular afferent responses, with relevance to understanding origins of myogenic reflex responses commonly used in the clinic to assess vestibular function, and vestibular short latency potentials commonly used for vestibular phenotyping in rodents.

## Results

### vCAP sensitivity with changes in rise-time

Primary striolar afferents and their myogenic counterpart, the VEMP, have been shown to be sensitive to the very onset of the stimulus envelope and are attenuated with increases in the stimulus rise-fall time^46^. However, the associated mechanical activation during vestibular afferent response generation under these conditions is unknown. To examine the stimulation sensitivity of the vestibular striolar afferents, vCAPs were monitored with simultaneous measures of macular epithelial vibration during changes in input voltage duration (or rise-time) across the various paradigms: Iso-Stimulus Voltage, Iso-Macular Velocity, Iso-Linear Acceleration, and Iso-Linear Jerk. The general approach was to examine the stimulation induced changes in the vCAP and associated mechanics in relation to the changes in various stimulus parameters.

### Iso-stimulus voltage

**Figure 2:**
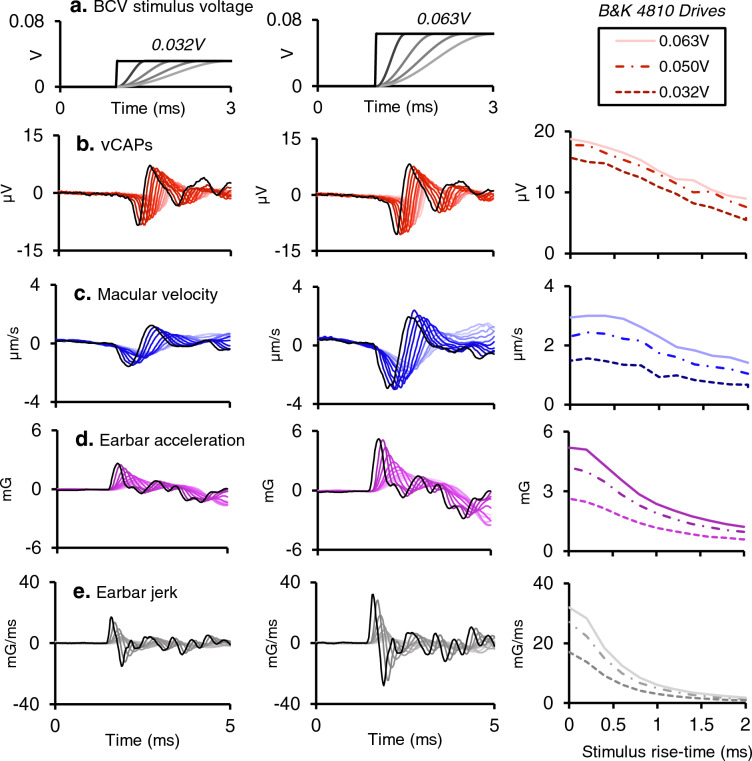
Simultaneous recordings of vCAPs, macular velocity, earbar acceleration and earbar jerk during Iso-Stimulus Voltage to the minishaker at different intensities, between 0.032 and 0.063 V. (**a**) The BCV voltage drive to the minishaker was kept constant whilst the stimulus rise fall-time was varied between 0 and 2 ms (0–50% stimulus waveform) for a 4 ms BCV monophasic pulse for 0.032 V (Left panel) and 0.063 V (Middle panel). Simultaneously measured responses include (**b**) vestibular compound action potentials (vCAPs) (red), (**c**) Laser Doppler vibrometry (LDV) measurements of utricular macular velocity recorded from a reflective microbead from the basal epithelial surface, (blue), (**d**) linear acceleration (magenta), and its derivative, (**e**) linear jerk (grey) recorded from a triaxial accelerometer coupled to the earbar near the skull. Responses in the left and middle panel correspond to the lowest (0.032 V) and highest (0.063 V) stimulus intensity, respectively. Peak-peak amplitudes for vCAPs, macular velocity, earbar acceleration, and earbar jerk are displayed in the right panel associated with changes in drive and stimulus rise-time.

**Figure 3:**
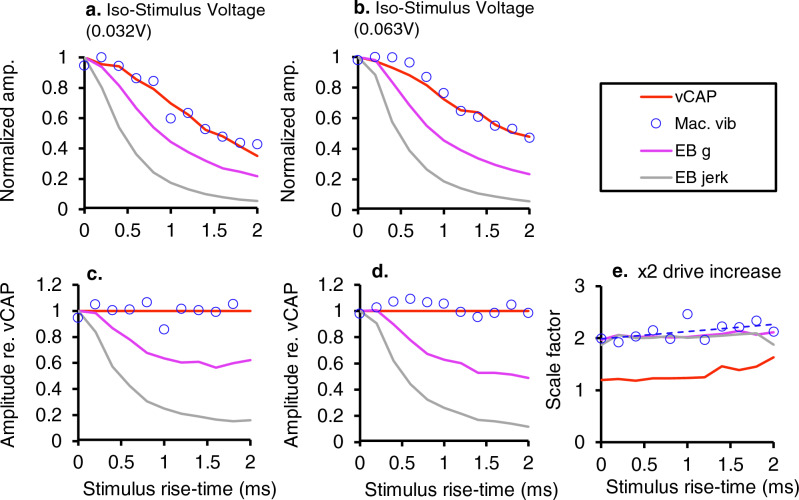
Normalized amplitudes and comparative scaling of vCAPs, macular velocity, earbar acceleration, and earbar jerk during BCV Iso-Stimulus Voltage (**a**, **b**). When the stimulus voltage to the minishaker was fixed (Iso-Stimulus Voltage) and rise-time was altered, vCAPs scaled closely with macular velocity. In terms of earbar kinematics, vCAPs scaled most closely with linear acceleration, rather than the first derivative, earbar jerk, for pulsatile BCV (**c**, **d**). Response parameter amplitudes normalized to vCAPs further emphasise macular velocity closely follows vCAP scaling during BCV across changes in stimulus rise-times, and vCAPs are more sensitive to acceleration rather than jerk (**e**). Response scaling associated with a doubling in BCV drive associated with changes in stimulus rise-time reveals vestibular afferents driving vCAPs have a compressive nonlinear scaling, whereas macular and earbar macromechanics have a passive and linear scaling.

The peak stimulus voltage supplied to the Bruel & Kjaer minishaker as a 4 ms square wave pulse was kept constant, whilst varying the stimulus rise-time between 0 and 2 ms (Fig. 2a). vCAPs, macular vibration, linear acceleration, and its derivative, linear jerk, were simultaneously measured. All responses declined as a function of stimulus (drive) rise-time, albeit at different rates (Fig. 2b–e). Normalizing the amplitude of each response to that of the shortest rise-time response revealed changes in vCAP amplitude (Fig. 3, red) were closely correlated with the changes in macular velocity (Fig. 3, blue circles). Both the vCAP amplitude and macular velocity declined proportionately with increases in rise-time for all stimulus intensities tested (Fig. 3a,b). By contrast, linear acceleration and linear jerk declined more rapidly with increased stimulus rise-time, with linear jerk displaying the greatest rate of decline (Fig. 3a,b). To further illustrate these relationships, response amplitudes were normalized to the vCAP amplitude (Fig. 3c,d).

Doubling the BCV input voltage drive (0.03 V vs 0.06 V) resulted in a doubling of the mechanical response sensitivity, which included macular velocity, linear acceleration, and linear jerk (Fig. 3e). By comparison, the same two-fold increase in BCV drive resulted in a compressive scaling of the vCAP (~ 1.2–1.5 × increase), suggesting vestibular neural output is saturating and nonlinear, whereas macular mechanics are linear and passive.

### Multiple iso-parameter comparisons

**Figure 4:**
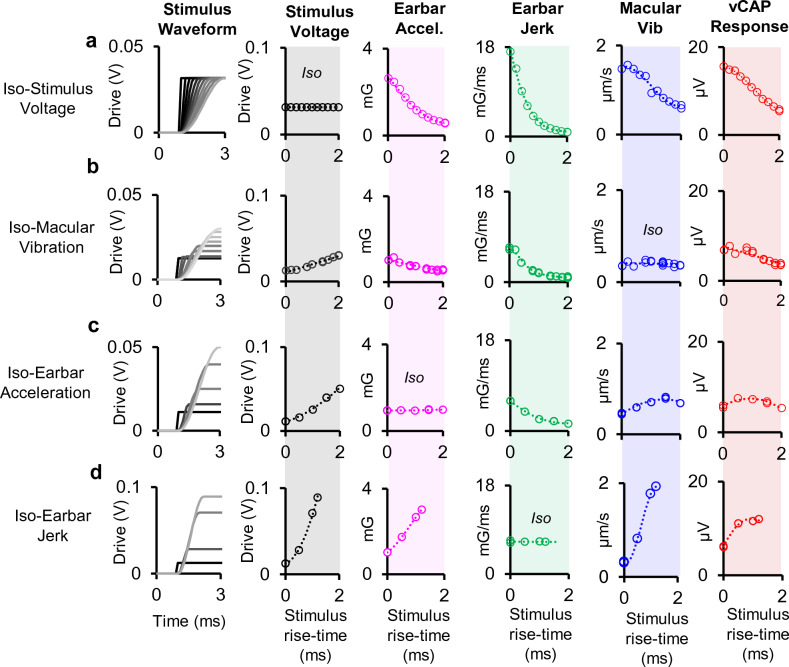
Multi-parametric comparisons of macular responses and input drives with changes in stimulus rise-time. Simultaneous measurements of stimulus voltage (grey), earbar acceleration (magenta), earbar jerk (green), macular velocity (blue), and vCAPs (red) associated with (**a**). Fixed peak voltage to minishaker (changes in rise time) or Iso-Stimulus Voltage. (**b**) Fixed peak macular velocity or Iso-Macular Velocity. (**c**) Fixed peak temporal bone acceleration or Iso-Linear Acceleration, and (**d**) Fixed peak temporal bone jerk or Iso-Linear Jerk.

To further investigate relevant kinematic elements of transient motion responsible for evoking synchronized vestibular afferent responses (or vCAPs) at the level of the macula and temporal bone (ear-bar), multiple iso-parameter response measures were compared, including Iso-Stimulus Voltage (Fig. 4a), Iso-Macular Velocity (Fig. 4b), Iso-Linear Acceleration (Fig. 4c), and, Iso-Linear Jerk (Fig. 4d). Results indicate that vCAP responses scale proportionately with macular velocity across all paradigms tested. For stimulation sensitivity related to the temporal bone (or ear-bar), vCAPs (and macular velocity) scale reasonably well with linear acceleration for short stimulus rise-times (< 1 ms) and begin scale with linear jerk for longer stimulus rise-times (> 1 ms). This is especially evident for Iso-Earbar Jerk (Fig. 4d).

### vCAP chirp sensitivity

**Figure 5:**
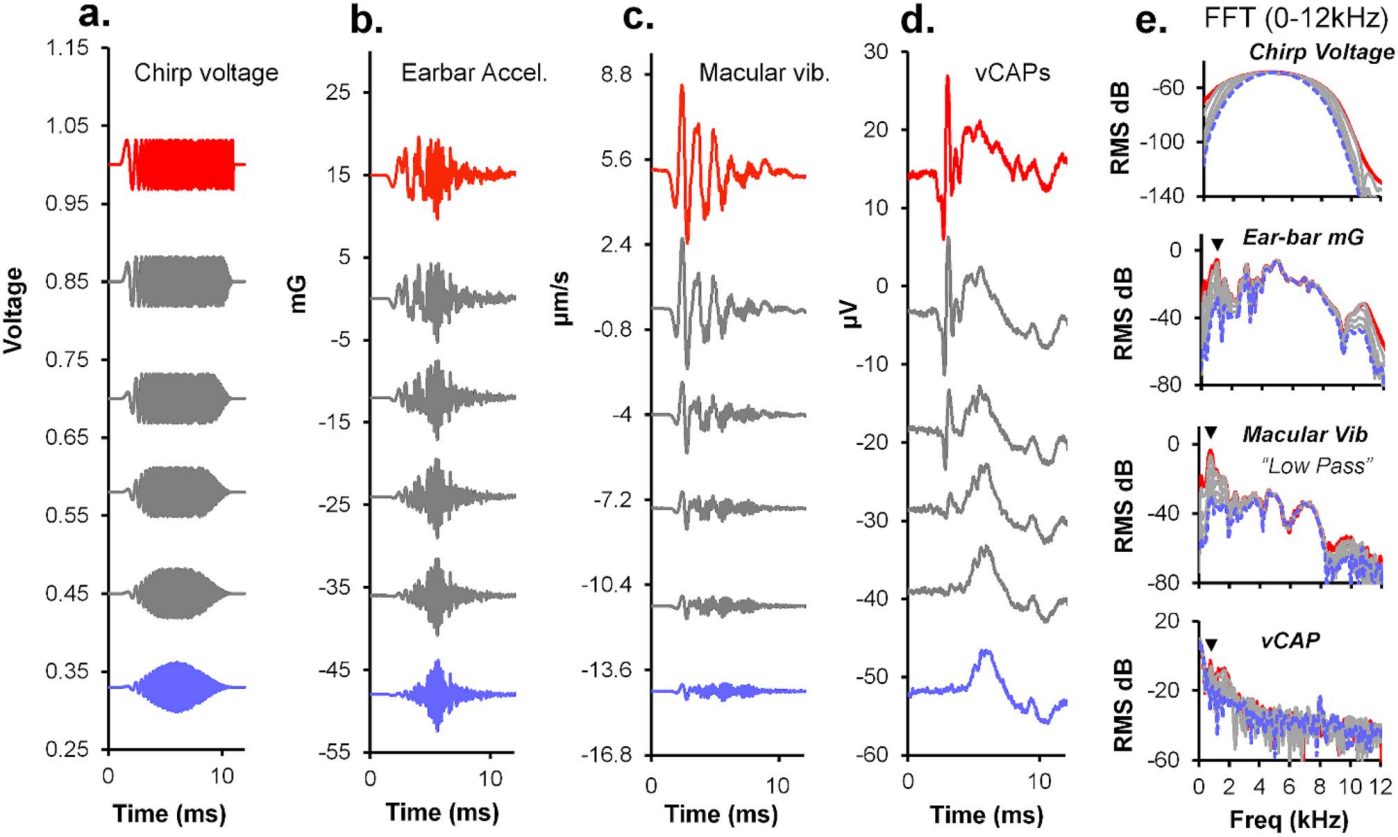
vCAP and macular sensitivity to broadband BCV chirps with changes in stimulus rise-time. (**a**) The rise-time of a 10 ms BCV Up-sweep chirp was varied from 0 and 5 ms (0–50% stimulus waveform) and simultaneous measurements of (**b**) linear acceleration, (**c**) macular velocity, and (**d**) vCAPs were recorded. (**e**) Associated FFT spectra (Hanning window) for waveforms displayed in a-d. Arrowheads show relevant frequency characteristics for generating synchronized vCAPs (spectra below 2 kHz).

A chirp is a broadband stimulus that can produce both increasing (up-chirp) or decreasing (down-chirp) frequency-variant shifts with time. Chirps are starting to be used more often in the clinic to evoke VEMPs^47^, however the adequate stimulus components associated with peripheral neural activation and biomechanics is unclear. BCV chirps were used here to assess the relationship between mechanical activation of the macula and vCAP generation for more complex vibrational stimuli. A 10 ms up-chirp (0 ms rise-time) (Fig. 5a) with a frequency range between DC to ~ 10 kHz, produced a ‘filtered’ acceleration of the ear-bar with several resonant peaks around 1, 3, and 5 kHz. This produced a band-limited vibration of the macula (Fig. 5c), with a dominant spectral peak around 1 kHz. These results indicate that high-frequency (> 2 kHz) temporal bone vibration does not result in high-frequency vibration of the macula, and that the macular biomechanics is relatively “Low Pass”. Smoothing the low-frequency onset of the up-chirp stimulus was performed to further examine relevant stimulus characteristics of the broadband stimuli. Changing chirp rise-time from 0 to 5 ms of a 10 ms stimulus waveform, completely abolishes the vCAP response, leaving behind a contralateral Auditory Brainstem Response (ABR), which disappears following contralateral cochlear ablation (data not shown). The ABR response scales closely, in timing and amplitude, with the mid-latency (high frequency) components of interaural acceleration (Fig. 5b), whereas the vCAP scales closely with onset ‘low-frequency’ macular velocity and interaural acceleration (Fig. 5b–d). Relevant frequency signatures for evoking the vCAP are denoted by the arrowheads in Fig. 5e. These results reveal that even high-frequency vibrational stimulus up to ~ 10 kHz, will only produce a ~ 1-2 kHz vibration of the macula, which is the relevant stimulus to generate synchronized otolithic afferent responses and sensory vCAPs.

**Figure 6:**
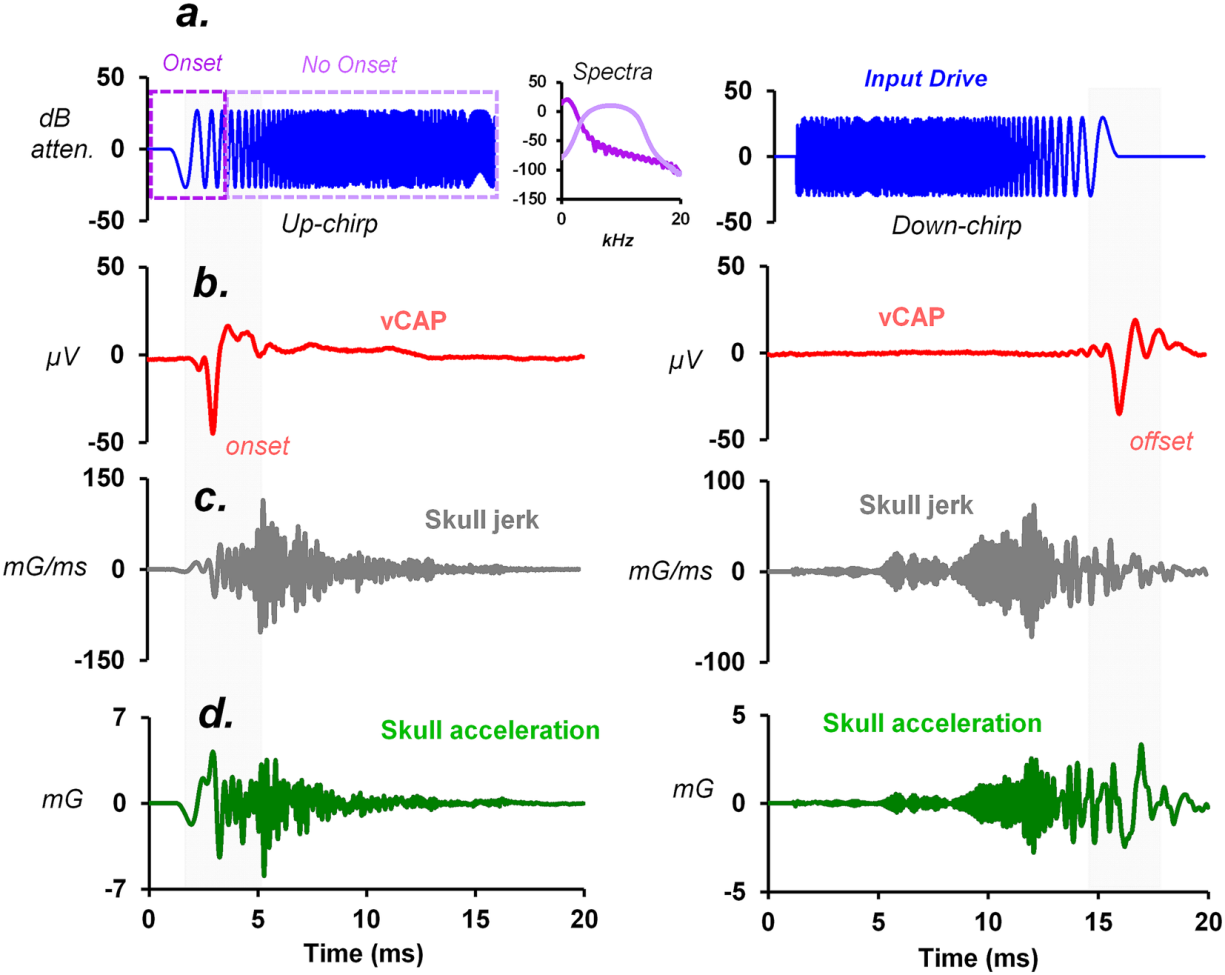
The relationship between chirp direction, vCAP response generation and latency. (**a**) Up-sweep versus down-sweep BCV chirps generated (**b**) vCAPs with similar amplitudes but with largely different latencies, which were temporally synced to the low-frequency component of the broadband stimulus. (**c**) Simultaneously measured skull jerk, and (**d**) skull acceleration recordings reveal the bandlimited onset or offset of the up- or down-sweep acceleration waveform is the relevant stimulus component to generate vCAPs, and not the higher-frequency components. Inset: Chirp stimulus power spectrum (Hanning window).

To further probe the relevant stimulus characteristics for evoking transient vestibular responses, both up-sweep and down-sweep chirps were used to evoke vCAPs, with corresponding measures of skull (ear-bar) vibration (Fig. 6a,d). The frequency of the chirp ranged from DC to 18 kHz, however, resonances of the skull-minishaker arrangement meant that skull vibration was not equal for all frequencies, with a particular reduction in the high-frequency components of the skull vibration (also see. Figure 5e). Data reveal that the vCAP is only evoked by the low-frequency component of the chirp stimulus, where most of the spectral power of the vibration was below 1 kHz (Fig. 6a). Here, vestibular receptor activation and vCAP generation is governed by the undamped low-pass biomechanics of the otoliths, with a natural frequency below 1 kHz.

### vCAP sensitivity with changes in frequency

**Figure 7:**
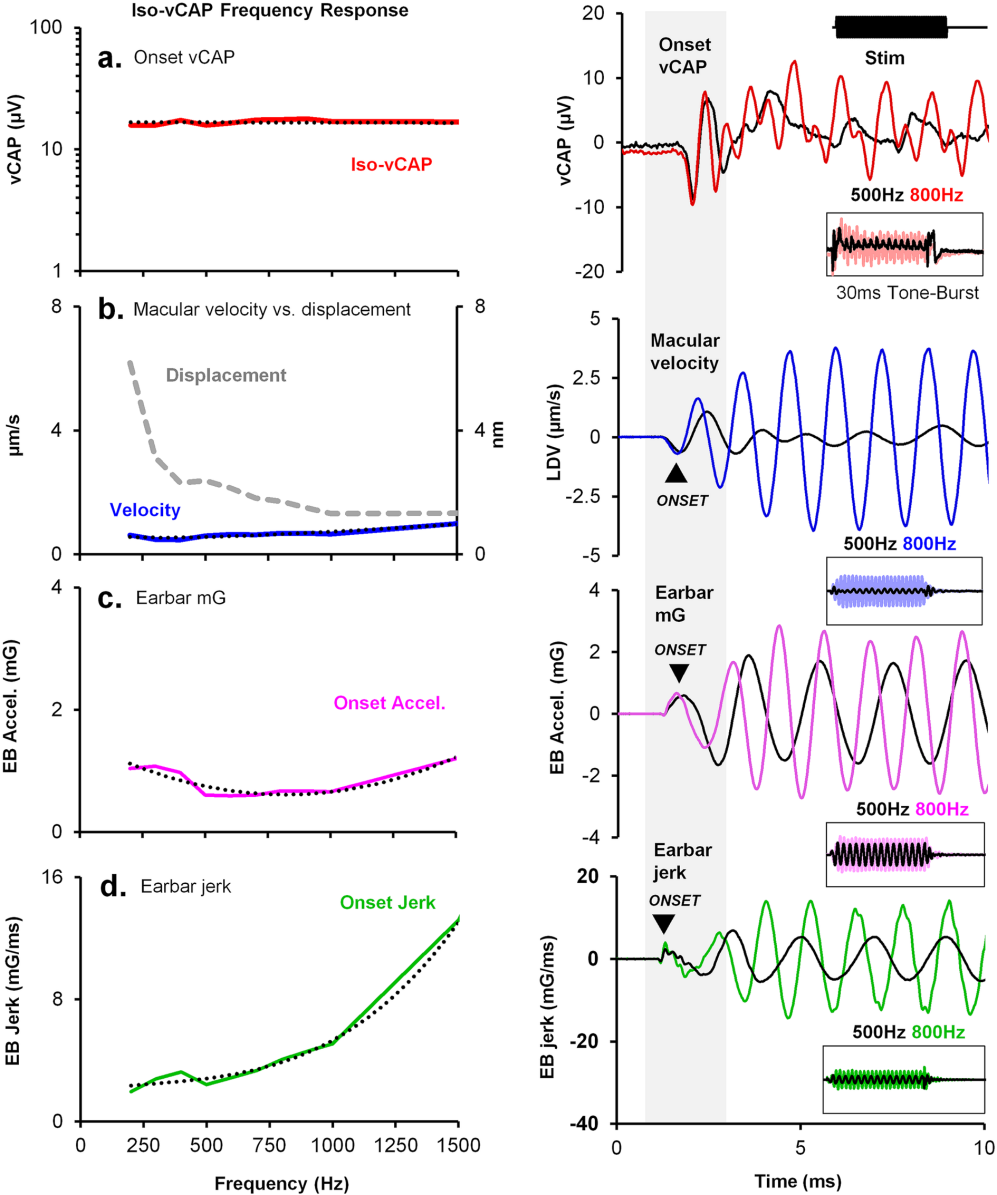
Iso-vCAP frequency response tuning curve. Left panel. (**a**) Onset vCAPs were kept constant during a 30 ms BCV tone burst (0 ms rise-time) across frequency (up to 1.5 kHz), with simultaneous measurements of (**b**) macular velocity, (**c**) linear acceleration, and d. its kinematic derivative, linear jerk. For a flat vCAP amplitude across frequency (Iso-vCAP), macular velocity also remained relatively flat, suggesting the primary afferents generating vCAPs are sensitive to macular velocity and not macular displacement for BCV tone-burst stimuli. In terms of cranial sensitivity, earbar acceleration changed by approximately a factor of ~ 0.3x, whereas earbar jerk changed by ~ 7x, suggesting vestibular afferent sensitivity is more likely to occur when acceleration is the main determinant, rather than kinematic jerk. Right panel. Representative waveform comparisons for the onset vCAP, macular velocity, linear acceleration, and linear jerk associated with a 500 Hz (black) and 800 Hz (coloured) tone-burst, respectively (10 ms window). Inset: Entire 50 ms time-domain window of the tone-burst response.

Tone bursts are routinely used in the neuro-otology clinic to evoke vestibular reflex responses, such as the VEMP, as a part of a standard assessment of otolith function. Although there is mixed data on VEMP tuning curves, likely due to differences across recording setups, 500 Hz is often used as the BCV impulse tone-burst frequency. However, there is mounting evidence that lower frequencies may be more effective for BCV activation of VEMPs. Despite this, the neurophysiological basis for VEMP tuning at the end-organ level is unclear relative to mechanical input and the generation of MET currents. To characterize the vCAP frequency response as a proxy from utricular afferent sensitivity across frequency, BCV tone bursts between 100 and 2000 Hz of varying intensity levels were used to evoke a fixed amplitude vCAP response, with simultaneous measures of epithelial vibration (Iso-vCAP frequency tuning curve; Fig. 7a). Associated macular velocity, macular displacement, linear acceleration, and linear jerk was also plotted against the frequency of the BCV stimulus (Fig. 7b–d). Results reveal that for an Iso-onset vCAP response (Fig. 7a), the associated onset macular velocity (taken as the initial N1 transient bump) remains relatively flat across frequency (Fig. 7a,b), suggesting that the vCAP scales with onset macular velocity for transient stimuli such as tone-bursts and pulses. By comparison, macular displacement declined exponentially with stimulus frequency, with displacement being largest at low frequencies. Linear acceleration approximated a parabolic function over frequency (Fig. 7c), whereas linear jerk increased exponentially (Fig. 7d). At low frequencies (< 450 Hz), linear jerk was relatively flat and had comparable scaling to the onset vCAP, consistent with the finding that vestibular afferents scale with jerk for spectral power below the natural frequency of the otoliths.

### VM frequency tuning curves

**Figure 8:**
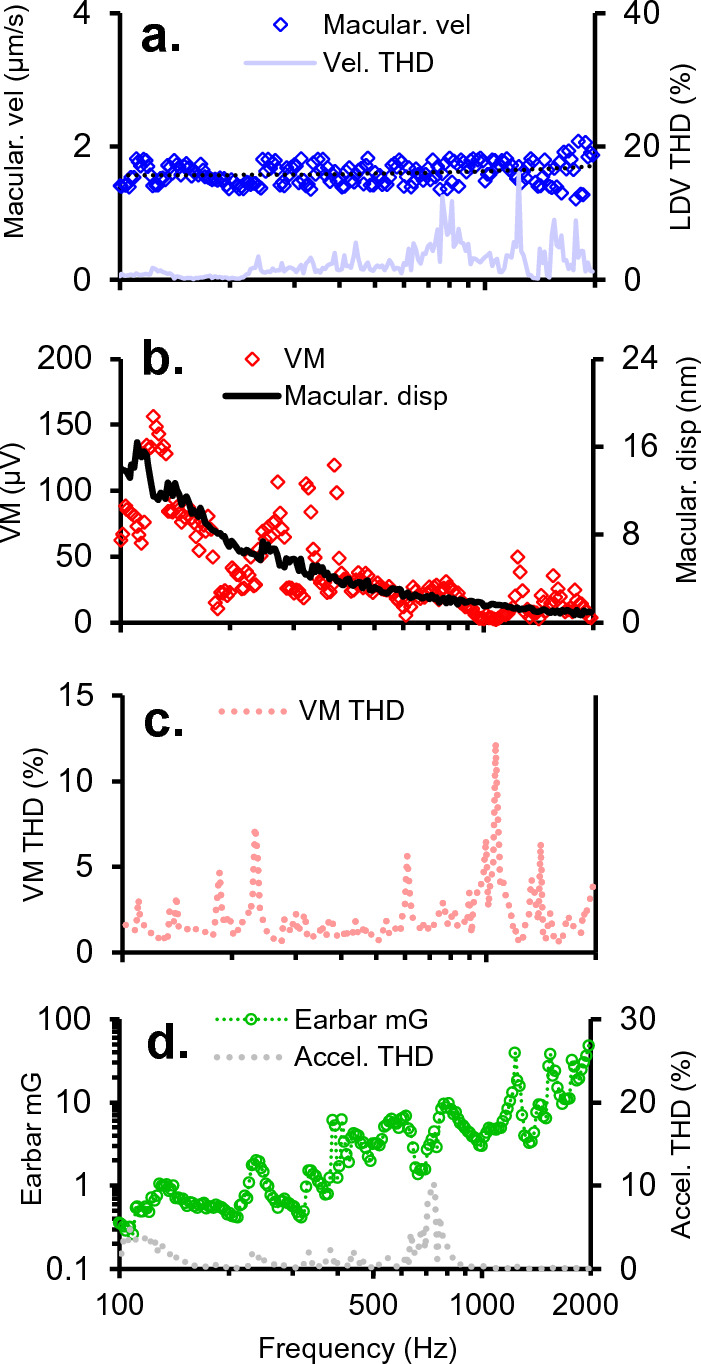
BCV vestibular microphonic (VM) frequency tuning curve. (**a**) Macular velocity (blue) measured via laser Doppler vibrometry (LDV), was kept constant for BCV stimuli between 100 and 2000 Hz (Iso-Macular Velocity), with simultaneous measurements of LDV total harmonic distortion (THD; light blue), (**b**) vestibular microphonics (VMs), macular displacement (taken as the integral of LDV macular velocity), (**c**) vestibular microphonic THD, (**d**) linear acceleration and the associated linear acceleration THD. In contrast to the vCAP (Fig. 7), results reveal that the VM increased in close proportion to macular displacement, indicating the net MET current entering hair cells proximal to the electrode was gated primarily by displacement and not velocity. Differences between hair cell and neural response dynamics reflect adaptation signal processing placed between the MET current and action potential generator in vestibular primary afferents.

To date, there has been no comprehensive in vivo mammalian data examining the origins of phasic signal processing in the vestibular calyx afferents in relation to presynaptic hair cell/mechanical sensitivity, and clinically relevant stimuli such as transient stimuli or frequencies above a few hundred Hz. This phasic signal processing is hypothesised to arise between the hair cell receptor potential and afferent spiking. However, another possibility is that the type-I VHCs are viscously coupled, which interposes a time derivate between the otoconial layer deflection and MET current. To explore this hypothesis, VMs were recorded across frequency with simultaneous recordings of macular velocity and linear acceleration. Voltage drives to the minishaker were programmatically altered to produce a fixed macular velocity across frequency from 100 and 2000 Hz (Fig. 8a), whilst simultaneously recording the VM, macular displacement, linear acceleration, and total harmonic distortion of the recording system (Fig. 8b–d). Results reveal that for a fixed macular velocity across BCV frequency (Fig. 8a), VM amplitude and sensitivity is closely correlated with macular displacement (Fig. 8b), and this tuning is independent of temporal bone acceleration and distortion in the recording setup (Fig. 8c–d).

## Discussion

Transient linear vibration stimuli such as hammer or finger taps^48^, brief BCV stimuli, and tone-bursts delivered by audiometric bone transducers are routinely used in the clinic or laboratory to evoke robust VEMP and vCAP responses. However, the mechanisms underlying these neurophysiological responses are not well understood at the end-organ level. In the present work, we directly measured mechanical vibration of the macula, VMs, and vCAPs in guinea pigs to determine how clinically relevant BCV stimuli evoke synchronized action potentials in the utricular nerve.

We first examined the relationship between the BCV stimulus and the vibration of the macula by comparing the peak macular velocity to the peak linear ear-bar acceleration (G) and jerk (G/s) for a series of stimulus strengths. Results in Fig. 4 and Supplementary Figs. 5, 6 and 7 demonstrate the peak macular velocity increases roughly in proportion to the input acceleration stimulus, consistent with the prediction of simple one degree-of-freedom (1-DOF) models of the utricle for stimuli at or below the corner frequency^49,50^. Present experimental LDV measurements demonstrate the sensory epithelium vibrates relative to the temporal bone. Hence, deflection of hair bundles involves at least 2 degrees of freedom, where the epithelium moves relative to the temporal bone and the otoconial layer moves relative to the surface of the sensory epithelium. Mechanical simulations using a 2-DOF model of utricular mechanics (a 4th order system) driven by BCV and ACS^51^ reproduce the LDV velocities reported here, including a switch from acceleration to jerk sensitivity with increasing frequency, further confirming that the utricle behaves as a simple inertial sensor that responds to acceleration transients. The present LDV measurements are consistent with a slightly underdamped mechanical response, exhibiting low-pass sensitivity to sinusoidal inter-aural vibration with a corner frequency near 500 Hz^51,52^.

In terms of the applied BCV stimulus, present results reveal the vCAP magnitude scales most closely with linear acceleration for short drive rise-times (< 1 ms), and switches to linear jerk for longer duration rise-times (> 1 ms). This switch is described in more detail in a complementary modelling paper^51^. These results were also reproduced across three experimental paradigms, which included Iso-Macular Velocity (Fig. 4b), Iso-Linear Acceleration (Fig. 4c), and Iso-Linear Jerk (Fig. 4d). For short rise-times, the vCAP magnitude scaled most closely with macular velocity, and linear acceleration, rather than other kinematic components such as macular displacement or linear jerk (or macular acceleration; not shown, or linear displacement; also, not shown). Hence, for brief BCV stimuli, linear acceleration of the temporal bone was the adequate stimulus to generate synchronized vCAPs in the present guinea pig experiments. Based on oVEMPs, the mechanical corner frequency in humans is probably about half that directly recorded here in guinea pigs^53^. If true, the transition from acceleration sensitivity to jerk sensitivity would be expected to occur in humans at a longer rise time of ~ 2 ms.

At longer stimulus pulse widths, vCAP scaling approximated the time-derivative of linear acceleration, which is consistent with previous VsEP experiments in rodents where linear jerk was identified as the adequate stimulus to generate evoked responses^40,45^. Despite this, there are key differences between the present report and previous VsEP studies that likely underlie the difference in sensitivity including: (1) Animal model: use of the guinea pig (*Cavia porcellus*) in the present report vs. mice (C57BL/6 J) or rats (Sprague Dawley) in previous VsEP experiments; (2) Stimulus: ~ 3mG inter-aural acceleration at ~ 20mG/ms in the present report vs. ~ 2000mG nasal-occipital acceleration at ~ 1000mG/s jerk in a supine position; (3) vCAP recording: non-inverting (active) electrode inserted in the facial nerve canal in the present report vs. scalp; (4) Surgical Approach: ablation of the cochlea in the present report vs. keeping the cochlea intact; (5) Anesthetics and medications: isoflurane vs. ketamine/xylazine, and the use of pre-anesthetics medications in the present report, such as opioids, i.e., buprenorphine, and mAChR antagonists, such as atropine, which may alter primary afferent or even efferent neuron sensitivity. Among all of these differences, a theoretical model of mechanical activation of the utricle by BCV and ACS^51^ suggests the primary determinant of acceleration vs. jerk sensitivity is the frequency content of the stimulus relative to the major corner frequency of the otolith organ in the direction stimulated. Stimuli below the corner are predicted to show jerk sensitivity, while stimuli near the corner are expected to show acceleration sensitivity. Therefore, differences between species in size of the utricle and differences between stimuli likely explain jerk vs. acceleration scaling of the vCAP. A broad-band stimulus would be expected to evoke more complex vCAPs that do not clearly scale with jerk or acceleration. For this reason, we use the term vCAP for compound action potentials evoked by any vestibular stimulus and reserve VsEP for vCAPs that scale with linear jerk. Macular velocity was not recorded in previous VsEP experiments but based on the present results we would expect the relationship between vCAP and macular velocity to hold even for stimuli where the VsEP scales with linear jerk.

Chirps are used to evoke cochlear responses in animal models and the clinic, such as the chirp-evoked ABR^54^. Special stimuli have been created to overcome travelling wave delays associated with cochlear mechanics^55^. Recent studies have extended these stimuli to the vestibular system to generate VEMPs^47,56^, however, it is unclear how these relatively complex stimuli evoke synchronous neural responses at the end-organ level. Moreover, many of the stimuli which have translated from the cochlea to the vestibular system have been designed to suit unique features of auditory transduction^57^. Hence, it is unclear how chirps are suited for otolithic receptor activation and how broadband stimuli vibrate the macula and activate otolithic hair cells. Present results reveal that chirps between the frequency range of DC and 10 kHz produce filtered temporal bone (ear-bar) acceleration with several resonant peaks and low-pass macular vibration with a dominant peak around 1 kHz, which is the adequate stimulus to generate sensory vCAPs. This means that the majority of the chirp signal is not making its way down to the macular epithelium to vibrate the mechanosensory hair cells. Data reveals vCAPs respond to the initial onset or offset peak of macular velocity (for up- and down-chirps, respectively), with relevant spectral power below 1 kHz (Figs. 5 and 6). When the transient onset (or offset) is smoothed by increasing the rise-time, the vCAP and macular vibration response sensitivity decreases (Fig. 5). These results provide a neurophysiological framework for earlier clinical findings, which reported robust VEMPs in humans evoked by band limited chirps (250–1000 Hz), chosen because of the purported sensitivity range of the otoliths^47,58^.

To determine how macular vibration is related to MET currents entering sensory hair cells, we compared the VM to the macular velocity and macular displacement for sinusoidal BCV tone bursts. The VM is the voltage modulation in the endolymph relative to reference ground measured adjacent to epithelium and reflects changes in the net MET current entering hair cells caused by hair bundle deflection. Results in Fig. 8 show the VM, and therefore the net MET current, is closely aligned with macular displacement over the entire bandwidth tested from 0.1–2 kHz. Results are consistent with the hypothesis that hair bundles are deflected primarily by otoconial layer displacement, not velocity, and that hair bundle shear is directly related to the macular displacement measured here using LDV^59^.

While the magnitude of VMs measuring the net MET currents scaled with macular displacement, the magnitude of vCAPs measuring the action potential synchronization scaled with onset macular velocity (Fig. 7). This difference highlights rate-sensitive signal processing occurring after the MET current^24^ manifests primarily as a time derivative in sensitive calyx bearing afferents that synchronize to transient stimuli. In terms of the clinically relevant BCV stimulus to generate synchronized vCAPs, results reveal the abrupt onset of the tone-burst is significant.

A potential practical implication of these findings relates to the frequency tuning of vestibular afferents to BCV tone-burst stimuli. Data suggest that vestibular afferent frequency tuning is likely associated with acceleration sensitivity, rather than when jerk is the main determinant. That is, for flat vCAP and macular velocity responses between 200 and 1500 Hz, onset jerk changes by almost a factor of 10, whereas onset acceleration remains relatively flat in comparison (Fig. 7).

Moreover, onset vCAPs scale with the initial transient component of the tone-burst stimulus (Fig. 7). This aligns with pulsatile and chirp BCV data, where vCAP and macular sensitivity is greatest for a 0 ms rise-time (Figs. 2 and 3). This is consistent with clinical data, where BCV oVEMP responses scaled with the initial stimulus onset waveform and showed no significant increase in amplitude with increasing stimulus duration^60^.

Overall, this work provides new insight into mechanical and neural mechanisms underlying synchronized action potential generation in sensitive mammalian calyx afferents in vivo. New findings from this work demonstrate that: 1. Synchronized irregular vestibular afferents are not universally sensitive to linear jerk, as previously thought. In the guinea pig, vCAPs scale with linear acceleration for brief stimuli (< 1 ms) and begin to mode switch, scaling with linear jerk for longer stimuli (> 1 ms). This mode dependence relies on mechanical factors controlling the dynamics of sensory hair cell activation, which was modelled in a complimentary paper^51^. In terms of clinical significance and stimulation modes, such as BCV tone-bursts and pulses, these data and associated theoretical work^51^ suggest vestibular afferents in humans are likely sensitive to linear acceleration, and not jerk. Here, the stimulus pulse width related to the mode switch (from acceleration to jerk sensitivity) increases (from 1 to 2 ms) for larger mammals (primates, humans, etc.) with a bigger utricle, compared to smaller mammals such as rodents with smaller utricles and less inertia^51^. Hence, most clinical stimuli in humans (0–2 ms pulse duration) will produce synchronized vestibular afferent responses which are predicted to scale with linear acceleration. These findings have potential implications for the design of stimulating parameters for activating otolith receptors in the clinic. Results suggest the optimal BCV stimulus for synchronizing vestibular afferents is a transient onset acceleration pulse (< 1 ms) of the temporal bone with 0 ms rise-time. This provides strong support for impulsive stimuli which are currently used by numerous groups (such as, the Type-4810, B81, and tendon-hammer)^39,48,61–63^. 3. Broadband BCV stimuli like chirps (up to 10 kHz) produce ‘low-pass’ vibration of the utricle (< 1–2 kHz). Hence, vestibular afferents generate a synchronous onset discharge in response to onset (or offset) mechanical stimulation with band-limited characteristics suited to the relatively low-pass biomechanics and natural frequency of the otoliths. 4. Phasic signal processing and velocity sensitivity in calyx vestibular afferents measured through the vCAP do not have mechanical/hair cell origins, confirmed by simultaneous VM and vibrometer recordings. Here, the vestibular microphonic scales with macular displacement, highlighting phasic vestibular signalling and velocity sensitivity in the calyx afferent arises post-MET current.

Although this work was the first to directly measure the vibration of the macula during synchronized vestibular afferent responses, there are several limitations which must be considered in the overall context of this work. 1. To directly record single-point LDV measurements from the utricle, the cochlea must be surgically ablated, resulting in fenestration and dehiscence for a clear optical recording path. Importantly, however, the latency of the vCAP did not change before or after cochlear ablation, suggesting minimal changes to vestibular afferent function during this procedure. A promising future direction will be to simultaneously record vestibular afferent function and biomechanics using non-invasive techniques, such as Optical Coherence Tomography, to better understand the dynamic response and functional output with the labyrinth intact. 2. The vCAP in our guinea pig model originates from the utricle, confirmed by selective end-organ ablation. Hence, we did not have the ability to also probe saccular function. To get a more comprehensive understanding of otolith activation regarding clinical stimulation modes, both utricular and saccular recordings should be the target of future studies. 3. The translational scope of these results with regards to the vestibular testing is limited by the guinea pig model and the overall differences to clinical work, such as the surgery, and the delivery and magnitude of BCV stimuli to the skull. For example, in the guinea pig, BCV is delivered to the ear-bar frames by the minishaker transducer rod at a magnitude of ~ 1 mG, whereas in the clinic, BCV is delivered to Fz or the mastoid process between ~ 0.1–1 G. To improve clinical relevance, future recordings could be taken with the labyrinth intact (with the cochlea chemically silenced or acoustically masked), with BCV stimulation across a broad dynamic range.

## Conclusion

This work sought to examine the relationship between macular macromechanics and vestibular action potential generation from irregular striolar afferents to improve our understanding of their stimulation sensitivity and tuning to clinical stimulation modes. Unlike previous studies, which characterized the operation of vestibular primary afferents relative to intense cranial acceleration, this work goes one step further and characterizes sensitive synchronous vestibular afferent responses (vCAPs) relative to macular epithelial vibration, hair cell VMs and their input drives. In contrast to vCAPs, hair cell VMs increased in proportion to macular displacement, indicating that the net MET current entering all hair cells was gated primarily by displacement, not velocity. The difference between VM and vCAP dynamics reflects adaptation signal processing interposed between the MET current and action potential generation in sensitive vestibular afferents^24,25^, and is the same process responsible for phase-locking of otolith afferent action potentials to audio frequency inputs^33^ and dynamic stimulation^64^. For brief BCV pulses (< 1 ms) and chirps used in the present study, macular velocities and vCAPs both increased in proportion to temporal bone acceleration. At longer duration BCV pulses, vCAPs began to increase in proportion to temporal bone jerk, which aligns with previous VsEPs measurements in rodents at lower stimulus frequencies and higher stimulus strengths relative to the present study^45^.

## Supplementary Information

Supplementary Information.

## Data Availability

The datasets used during the current study, as well as the code for data acquisition and analysis are available from the corresponding author on reasonable request.

## Abbreviations

ACS: Air conducted sound
BCV: Bone-conducted vibration
LDV: Laser doppler vibrometry
MET: Mechanoelectrical transduction
vCAP: Vestibular compound action potential
VEMP: Vestibular evoked myogenic potential
VM: Vestibular microphonic
VsEP: Vestibular short-latency evoked potential
