# Supplementary material for: Using macular velocity measurements to relate parameters of bone conduction to vestibular compound action potential responses
Source: Sci Rep. 2023 Jun 23;13:10204. doi: 10.1038/s41598-023-37102-3 (PMC10290084; doi:10.1038/s41598-023-37102-3)
Supplement: Supplementary file 1 — Supplementary Information. [file 41598_2023_37102_MOESM1_ESM.docx]

**Supplementary Information (SI)**

**Using Macular Velocity Measurements to Relate Parameters of Bone Conduction to Vestibular Compound Action Potential responses.**

*Christopher J. Pastras^1,2*^, Ian S. Curthoys^3^, Richard D. Rabbitt^4#^, Daniel J. Brown^5#^.*

**Affiliations**

**^1^**Faculty of Science and Engineering, School of Engineering, Macquarie University, Sydney, NSW, 2109, Australia.

**^2^**School of Medical Sciences, The University of Sydney, Sydney, NSW, 2050, Australia.

**^3^**Vestibular Research Laboratory, The University of Sydney, School of Psychology, Sydney, NSW, 2050, Australia

**^4^**Departments of Biomedical Engineering, Otolaryngology and Neuroscience Program, University of Utah, Salt Lake City, UT, 84112, USA.

**^5^**School of Pharmacy and Biomedical Sciences, Curtin University, Bentley, Western Australia 6102, Australia

*Correspondence: [christopher.pastras@mq.edu.au](mailto:christopher.pastras@mq.edu.au)

**Supplementary Note 1.** Vestibular Compound Action Potentials were evoked by transient pulses (<1ms) or tone-burst stimuli (e.g., 800Hz). Neural origins of vCAP responses were validated following perfusion of Tetrodotoxin (TTX; 100µM in artificial perilymph; Sigma Aldrich, AUS) into the perilymphatic space at the basal epithelium of the utricular macula.

**Supplementary Figure 1 (S1).** Vestibular striolar primary afferents fire synchronized action potentials at short latencies and precise phase angles relative to input BCV pulses or train bursts, respectively. A. 0.5ms BCV pulses (0.25ms rise-fall) generate B. ear-bar acceleration transients which evoke robust C. vestibular nerve Compound Action Potentials (vCAPs, averaged; 100 presentations). The neural origin of vCAPs was validated by their chemical ablation following the perfusion of TTX (red line). D. 800Hz sinusoidal BCV results in a E. high-frequency acceleration burst, and a F. volley of extracellular vCAPs, also referred to as the Vestibular Nerve Neurophonic.

**Supplementary Note 2.** Laser Doppler Vibrometer was calibrated prior to each experiment. Here, the laser beam from the LDV was focused onto the tri-axial accelerometer, which was vibrated by a Brüel & Kjær minishaker. LDV velocity measurements were made in the same plane as the triaxial accelerometer (in the left-right or x-axis), and accelerometer output was integrated to velocity to validate LDV system output. LDV carrier delay was corrected programmatically on LabVIEW. Code can be made available from the corresponding author upon reasonable request.

**Supplementary Figure 2 (S2).** Calibration of LDV output. a. Schematic of the experimental setup used to calibrate LDV velocity against accelerometer velocity. b. Representative LDV velocity and earbar acceleration waveforms following Brüel & Kjær (B&K) minishaker stimulation (0.5ms vibration pulse). Accelerometer G integrated to velocity matches the waveshape and magnitude of LDV velocity, suggesting accurate calibration. c. Results of the 1.2ms LDV carrier delay correction across frequency.

**Supplementary Note 3.** When recording vestibular responses, attempts were made to position the bead at the central dark band in the middle of the macula, which corresponds approximately to the striolar zone of the neuroepithelium (Fig. 1 & 3). However, due to the delicate nature of the surgery, and with attempts to avoid contact/damage to the vestibular macula, bead positions varied subtly across animals. Measurements were undertaken to determine the effect of this variation. Recordings of macular vibration at the lateral striolar region revealed minimal differences to that of the central ‘striolar’ zone for brief vibration. This suggested that discrepancies in bead placement across animals did not alter mechanical results based on spatial tuning of the macula. Comparisons of temporal bone vibration were also compared to that of the macula, to quantify BCV transmission through the cranium to the macula.

**Supplementary Figure 3 (S3).** Dynamic response of the macula vs. bone to pulsatile vibration. A. Reflective microbeads were placed on the central (*green*) and lateral (*red*) macular region, and bone within the vestibule (*blue*), for LDV and accelerometer measurements during BCV. B. Representative LDV and accelerometer waveforms corresponding to 0.1V input drive to the minishaker. C. LDV Input-Output functions across BCV input drives corresponding to central and lateral macula and bone positions, for negative (dashed lines) and positive (solid lines) polarity stim. D. LDV response sensitivity relative to ear-bar acceleration in units of µm/s/mG, across input drives associated with different bead positions.

**Supplementary Note 4.** When recording vestibular responses researchers typically calibrate input stimuli using an accelerometer placed on various fixtures, such as headframes, bolts, or headbands. These locations are non-invasive and near the skull, providing a relatively accurate measure of input acceleration delivered to the temporal bone. In this study, linear acceleration was recorded from an accelerometer mounted to the ear-bar frame adjacent to the cranium. Recordings were performed to compare ear-bar acceleration to adjacent temporal bone acceleration measured using laser Doppler vibrometry. Results reveal ear-bar acceleration provides a good proxy for nearby temporal bone acceleration for transient and sinusoidal BCV in our experimental setup.

**Supplementary Figure 4 (S4).** Ear-bar vs. skull acceleration. a. Simultaneous measurements of vCAPs, temporal bone acceleration (recorded via LDV from the bone above the facial nerve canal) and ear-bar acceleration (recorded via a tri-axial accelerometer) b. reveal acceleration magnitude and latency recorded from the ear-bar and adjacent skull (evoked by a 0.5ms BCV pulse) are equivalent. The blue shaded area represents the section of the waveform used to calculate pk-pk acceleration. c. Comparisons of LDV temporal bone acceleration and ear-bar accelerometer driven by 200Hz, 400Hz, and 900Hz sinusoidal BCV stimulation, reveal R² values of 0.9988, 0.9997, and 0.9928, respectively, demonstrating high correlation.

**Supplementary Note 5.** Iso-Macular Vibration. Here, the stimulus voltage driving the minishaker was altered across rise times, to produce a fixed macular velocity response (using the amplitude of the first negative peak, N1, as a metric).

**Supplementary Figure 5 (S5).** Iso Macular Vibration and associated response waveforms and magnitudes. A. The magnitude of macular vibration (blue) was kept constant with changes in stimulus rise-time (0-50%; 0-2ms) associated with a 4ms BCV pulse. B. Its integral, macular displacement (grey) was quantified, along with C. synchronized vCAPs (red) recorded from the facial nerve canal, D. ear-bar acceleration (magneta), E. its integral, ear-bar velocity (cyan), and F. ear-bar jerk (green

**Supplementary Note 6.** Iso-Earbar Acceleration. To further probe the kinematic sensitivity of the vCAP with regards to the ear-bar (and cranium), vCAPs were recorded while keeping ear-bar acceleration constant as stimulus rise-time was varied.

**Supplementary Figure 6 (S6).** Iso-Earbar Acceleration and associated response waveforms and magnitudes. A. The magnitude of ear-bar acceleration (magenta) was kept constant with changes in stimulus rise fall time (0-50%; 0-2ms) associated with a 4ms BCV pulse. B. Its integral, ear-bar velocity (cyan), and C. its derivative, ear-bar jerk (green) was quantified, along with D. vCAPs (red), E. macular velocity (blue), and F. its integral, macular displacement (grey).

**Supplementary Note 7.** Iso-Earbar Jerk. To further examine the kinematic elements for generating synchronized vestibular afferent responses, vCAPs were recorded while keeping ear-bar jerk fixed as stimulus rise-time was varied.

**Supplementary Figure 7 (S7).** Iso-earbar jerk and associated response waveforms and magnitudes. A. The magnitude of ear-bar jerk (green) was kept constant with changes in input drive rise fall time (0-2ms) associated with a 4ms BCV pulse. B. vCAPs were measured from the facial nerve canal, (red), alongside C. ear-bar acceleration (magenta), D. ear-bar velocity (cyan), E. macular velocity (blue), and F. its integral, macular displacement (grey).
